# EMA and FDA psychiatric drug trial guidelines: assessment of guideline development and trial design recommendations

**DOI:** 10.1017/S2045796021000147

**Published:** 2021-04-30

**Authors:** Kim Boesen, Peter C. Gøtzsche, John P. A. Ioannidis

**Affiliations:** 1 Nordic Cochrane Centre, Rigshospitalet, Dept. 7811, Copenhagen, Denmark; 2 Meta-Research Innovation Center at Stanford (METRICS), Stanford University, Stanford, CA, USA; 3 Meta-Research Innovation Center Berlin (METRIC-B), Berlin Institute of Health, Charité Universitätsmedizin, Berlin, Germany; 4 Institute for Scientific Freedom, 2970 Copenhagen, Denmark; 5 Departments of Medicine, of Epidemiology and Population Health, of Biomedical Data Science, and of Statistics, Stanford University, Stanford, CA, USA

**Keywords:** Conflicts of interest, drug approval process, psychiatric drugs, randomised clinical trials, regulatory science

## Abstract

**Aims:**

The European Medicines Agency (EMA) and the US Food and Drug Administration (FDA) produce guidelines for the design of pivotal psychiatric drug trials used in new drug applications. It is unknown who are involved in the guideline development and what specific trial design recommendations they give.

**Methods:**

Cross-sectional study of EMA Clinical Efficacy and Safety Guidelines and FDA Guidance Documents. Study outcomes: (1) guideline committee members and declared conflicts of interest; (2) guideline development and organisation of commenting phases; (3) categorisation of stakeholders who comment on draft and final guidelines according to conflicts of interest (‘industry’, ‘not-industry but with industry-related conflicts’, ‘independent’, ‘unclear’); and (4) trial design recommendations (trial duration, psychiatric comorbidity, ‘enriched design’, efficacy outcomes, comparator choice). Protocol registration https://doi.org/10.1101/2020.01.22.20018499 (27 January 2020).

**Results:**

We included 13 EMA and five FDA guidelines covering 15 psychiatric indications. Eleven months after submission, the EMA had not processed our request regarding committee member disclosures. FDA offices draft the Guidance Documents, but the Agency is not in possession of employee conflicts of interest declarations because FDA employees generally may not hold financial interests (although some employees may hold interests up to $15,000). The EMA and FDA guideline development phases are similar; drafts and final versions are publicly announced and everybody can submit comments. Seventy stakeholders commented on ten guidelines: 38 (54%) ‘industry’, 18 (26%) ‘not-industry but with industry-related conflicts’, six (9%) ‘independent’ and eight (11%) ‘unclear’. They submitted 1014 comments: 640 (68%) ‘industry’, 243 (26%) ‘not-industry but with industry-related conflicts’, 44 (5%) ‘independent’ and 20 (2%) ‘unclear’ (67 could not be assigned to a specific stakeholder). The recommended designs were generally for trials of short duration; with restricted trial populations; allowing previous exposure to the drug; and often recommending rating scale efficacy outcomes. EMA mainly recommended three arm designs (both placebo and active comparators), whereas FDA mainly recommended placebo-controlled designs. There were also other important differences and FDA's recommendations regarding the exclusion of psychiatric comorbidity seemed less restrictive.

**Conclusions:**

The EMA and FDA clinical research guidelines for psychiatric pivotal trials recommend designs that tend to have limited generalisability. Independent and non-conflicted stakeholders are underrepresented in the guideline development. It seems warranted with more active involvement of scientists and independent organisations without conflicts of interest in the guideline development process.

## Background

Reports have found that newly authorised medicines often have questionable or no added patient-relevant benefits compared to older treatments (Motola *et al*., 2006; van Luijn *et al*., 2010; Prescrire, 2019; Wieseler *et al*., 2019; Erhel *et al*., 2020). In specialities like oncology, this seems to be particularly prevalent (Kim and Prasad, 2015; Davis *et al*., 2017; Gyawali *et al*., 2019; Naci *et al*., 2019). In a cohort of drugs approved by the German drug regulatory agency between 2011 and 2016, the Institute for Quality and Efficiency in Health Care (IQWiG) judged that 10% had substantial benefits compared to already available treatments, while 58% had no proof of added benefits (Wieseler *et al*., 2019). Out of 18 newly approved drugs in the combined field of psychiatry/neurology, only one was judged to have substantial added benefits compared to already available treatments (Wieseler *et al*., 2019).

Systematic reviews of commonly used psychiatric drugs, particularly of antidepressants for depression (Jakobsen *et al*., 2017; Munkholm *et al*., 2019) and of central stimulants for attention deficit hyperactivity disorder (ADHD) (Storebø *et al*., 2015; Punja *et al*., 2016; Castells *et al*., 2018; Cândido *et al*., 2021), have highlighted potential problems with low generalisability of psychiatric drug trials. Methodological limitations include small sample sizes, short trial durations, restricted trial populations in terms of allowed psychiatric comorbidity, risk of withdrawal effects due to previous exposure to the drug of interest, and use of surrogates and rating scales rather than patient-relevant outcomes.

Researchers have advocated for psychiatric pivotal trials, i.e. clinical trials conducted with the purpose of obtaining a marketing authorisation for a new drug or a new indication, with active comparators (i.e. already approved medications instead of placebo only) and a focus on hard, functional outcomes such as hospitalisation admissions and suicide rather than symptom rating scales (Barbui *et al*., 2007; Barbui and Garattini, 2007; Barbui and Bighelli, 2013). An assessment of the evidence base for new psychiatric drug approvals in Europe categorised the general evidence as being ‘poor’ (Erhel *et al*., 2020), and the European Medicines Agency (EMA)'s guidelines for designing pivotal psychiatric drug trials have been highlighted as important to improve the usefulness of these trials (Barbui and Bighelli, 2013). On a more general note, it has been argued that the threshold for new drug approvals has been lowered to accommodate the pharmaceutical industry's interests (Davis *et al*., 2016).

**Fig. 1.**
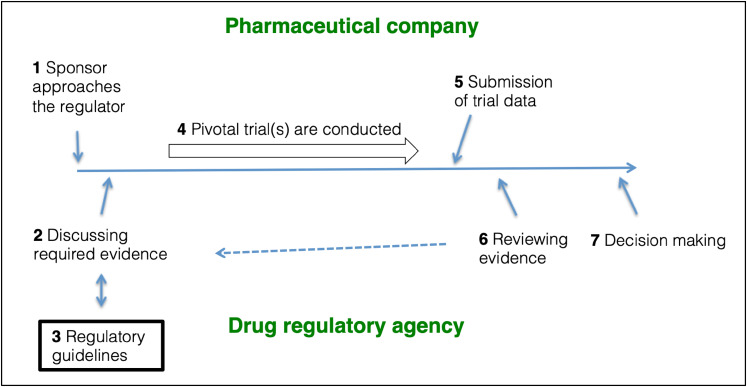
The role of regulatory guidelines in pivotal trial designs. (1) The sponsor approaches the relevant drug regulator with the purpose of getting a new drug authorised. (2) Sponsor and regulator discuss (and often agree) on a drug development program. (3) The design and conduct of the pivotal trials will often follow design recommendations outlined in regulatory guidelines. (4) The sponsor conducts the pivotal trials. 5) The acquired data are submitted to the regulators as a Marketing Authorisation Application (EMA) or a New Drug Application (FDA) using the Common Technical Format. (6) The regulator reviews the data, and, if necessary, steps 2, 3 and 4 are repeated to obtain additional information. (7) The regulator decides whether to authorise or reject the application. Figure based on FDA (2019*d*); EMA (2021*b*).

Pivotal trials are often designed in a bilateral agreement between the pharmaceutical company and the drug regulator (Fig. 1). The EMA and the US Food and Drug Administration (FDA) publish guidelines on how to design and conduct these pivotal trials for new drug approvals and these guidelines offer a roadmap in supporting the design of pivotal trials. EMA publishes ‘Clinical Efficacy and Safety Guidelines’ (EMA, 2020*a*), which the agency ‘strongly encourages’ their applicants to follow. These guidelines ‘reflect a harmonised approach of the EU Member States and the Agency on how to interpret and apply the requirements for the demonstration of quality, safety and efficacy [*…*]’ (EMA, 2020*b*). The FDA publishes ‘Guidance Documents’ (FDA, 2021). These documents are ‘intended to assist the pharmaceutical industry’ in designing and conducting pivotal trials, but they are not ‘legally binding or enforceable’ (FDA, 2005). The documents ‘represent current Agency thinking, sponsor submissions that conform to current guidance should be considered acceptable’ (FDA, 2005). Pharmaceutical companies and regulatory agencies may communicate at any stage of medicines development, e.g. EMA provides ‘scientific advice’ (EMA, 2021*a*).

Some of EMA's trial design recommendations (choice of comparator, outcomes, and patient population) for psychiatric drug trials have previously been described (Barbui and Bighelli, 2013). However, the development and content of EMA and FDA guidelines for psychiatric drug trials have never been systematically assessed. We wanted to assess (1) the guideline committee members and their declared conflicts of interest; (2) the organisation of commenting phases on draft and concept papers; (3) the stakeholders who comment on guideline draft and concept papers; and (4) the EMA and FDA design recommendations for five trial characteristics in pivotal psychiatric drug trials.

## Methods

### Inclusion of drug regulatory agency guidelines

We applied the following inclusion criteria: (1) the guideline (draft or final version) should be published by the EMA or FDA on how to design pivotal trials for drug approval; (2) the guideline should cover all aspects of pivotal trial design (i.e. what the FDA calls ‘umbrella guidance’) and not address specific trial aspects only, e.g. inclusion criteria or specific outcome measures; and (3) the guideline should cover psychiatric diagnoses as defined in current or previous versions of the *Diagnostics and Statistics Manual of Mental Disorders* (DSM) or the *International Classification of Diseases* (ICD). We included the newest guideline (draft or final version) if more than one guideline was published on the same subject. See eMethods for details.

### Guideline committee members' conflicts of interest

The guideline committee members and/or EMA and FDA employers that are responsible for drafting and revising the guidelines are not publicly disclosed. Similarly, the committee members' declared conflicts of interest are not publicly available. We therefore filed Freedom of Information Act requests to the EMA and FDA to access the committee member lists and their declared conflicts of interest. We wanted to report the proportion of committee members with declared conflicts of interest for each guideline.

### Guideline development

We assessed the development of draft guidelines in regards to, (1) public availability of draft versions for commenting; (2) active recruitment of specific stakeholders to comment on drafts; (3) applied restrictions on who may comment on the documents; (4) announcement of drafts and final guidelines, e.g. on social media, in newsletters or in relevant journals.

We searched the EMA and FDA websites for information. It was not necessary to contact the regulators for additional information. See eMethods for details.

### Stakeholder comment documents

EMA publishes dedicated documents with stakeholder comments submitted on draft guidelines along the final guidelines. The stakeholder comments and EMA's corresponding replies are collated in a single document and made available for each guideline (EMA, 2020*a*).

FDA manages and operates public correspondence on federal regulations and other rule-making documents through the *Docket's Management* (US Government, 2020). All official US Federal documents, such as drafts or final Guidance Documents, have their own designated entry, called a *Docket Folder Summary*, where the public can submit and also view submitted comments on the Guidance Documents.

For each included stakeholder document related to a specific guideline, we counted the total number of stakeholders, total number of comments, and total number of comments by each stakeholder. We categorised the stakeholders according to their conflicts of interest as ‘industry’ (e.g. pharmaceutical companies or other industry), ‘not-industry but with industry-related conflicts’ (e.g. organisations, associations or individuals reporting financial conflicts of interest related to pharmaceutical companies), ‘independent’ (organisations, associations and individuals with declared no conflicts of interest related to the pharmaceutical industry) or ‘unclear financial relationship’ (there was insufficient information to identify and/or categorise the stakeholder). We searched for information on Google, PubMed, OpenPaymentsData, and stakeholder websites regarding funding, annual budgets, and disclosed conflicts of interest. For organisations and associations, we focused on board and executive members. We did not define a lower monetary limit for conflicts of interest. See eMethods for details.

### Trial design recommendations

For all included research guidelines, we extracted recommendations regarding five trial design characteristics: (1) duration of follow-up, also after the randomised phase ended; (2) exclusion criteria related to psychiatric comorbidity; (3) allowing previous exposure to the drug or drug class, which may be called an ‘enriched design’ or ‘enrichment strategy’ (FDA, 2019*a*); (4) efficacy outcomes (primary and secondary); and (5) choice of comparator (e.g. placebo, active comparator, or both).

We wanted to put the trial design recommendation in a clinical context for each of the psychiatric diagnoses and corresponding guidelines, i.e. what is the natural history of the different conditions in terms of expected symptom duration; what are the most common comorbid psychiatric diagnoses and how common are they; and whether previous exposure to the drug might introduce potential withdrawal effects and also bias the sample of participants in favour of those who tolerate the drug. We extracted this basic clinical epidemiology information from the guidelines, when available. We further searched the Core Outcome Measures in Effectiveness Trials (COMET) database (COMET, 2020) for standardised sets of outcome measures for the individual indications, and we assessed the most recent systematic review (Eiring *et al*., 2015) of patient preferences for outcome measures in psychiatric drug trials.

See the Supplementary material for differences between the protocol (Boesen *et al*., 2020) and the final manuscript.

## Results

### Overview of results

We searched the EMA and FDA websites in February 2020 and identified 13 eligible EMA Clinical Efficacy and Safety Guidelines and five FDA Guidance Documents (Table 1). The 13 EMA guidelines were published between 2005 and 2017; the panic disorder guideline could not be downloaded. All EMA guidelines were adopted (i.e. final) versions, two (insomnia and schizophrenia) had been revised once, and the depression guideline had been revised twice. Concept papers for upcoming revisions were published for the depression and bipolar disorder guidelines. The five FDA guidelines were published between 2015 and 2019; four were draft versions and one (opioid use disorder) was a final guideline. The 18 guidelines covered 15 unique indications (depression, ADHD, and alcohol dependence were covered by both agencies). The full dataset is available here (https://osf.io/3xcdu/?view_only = 957edc6293894497ba7aaf5bb8fc8205).

**Table 1.** Overview of included regulatory research guidelines

Regulator	Condition	Guideline ID	Year of publication	Draft or final guideline
EMA	Autism spectrum disorder	EMA/CHMP/598082/2013	2017	Final
EMA	Depression	CHMP/185423/2010 Rev. 2	2013	Final (revision 2)^a^
EMA	Schizophrenia	CHMP/40072/2010 Rev. 1	2012	Final (revision 1)
EMA	Insomnia	CHMP/16274/2009 Rev. 1	2011	Final (revision 1)
EMA	Premenstrual dysphoric disorder	CHMP/607022/2009	2011	Final
EMA	Attention deficit hyperactivity disorder	CHMP/EWP/431734/2008	2010	Final
EMA	Alcohol dependence	CHMP/EWP/20097/2008	2010	Final
EMA	Post-traumatic stress disorder	CHMP/EWP/358650/2006 Corr. 2	2008	Final
EMA	Social anxiety	CHMP/EWP/3635/2003	2006	Final
EMA	Obsessive-compulsive disorder	CHMP/EWP/4279/2002	2005	Final
EMA	Generalised anxiety disorder	CPMP/EWP/4284/2002	2005	Final
EMA	Panic disorder	CHMP/EWP/4280/2002	2005	Final^b^
EMA	Bipolar disorder	CPMP/EWP/567/1998	2001	Final^a^
FDA	Attention deficit hyperactivity disorder	2019-09193	2019	Draft
FDA	Opioid use disorder	FDA-2018-D-1334	2019	Final
FDA	Major depressive disorder	FDA-2018-D-1919	2018	Draft
FDA	Low sexual interest, and/or arousal in women	No ID	2016	Draft
FDA	Alcoholism	FDA-2015-D-0152	2015	Draft

Listed in order of publication year.

a A concept paper for a revision of the guideline has been published.

b The link did not work so we could not include the guideline. Links to the guidelines can be found in Supplement 1.

### Guideline committee members' conflicts of interests

The EMA informed us they were not able to respond within their regular response time caused by EMA's relocation to Amsterdam. After 11 months from submission, EMA had not processed our request of access to committee member lists. See correspondence with EMA (Supplement 2).

The FDA Freedom of Information Team informed us during a telephone meeting that, (1) federal employees are not allowed to have financial conflicts of interest; (2) Guidance Documents are authored internally in the Agency by divisions or offices and not by individual employees; and (3) therefore the FDA is not in possession of conflicts of interest declarations related to Guidance Document development (see summary of the meeting in Supplement 1). We partly confirmed statement (1) from FDA's website (FDA, 2017*a*), where it is stated that FDA employees are generally prohibited from holding financial interests, e.g. stocks, in any FDA ‘significantly regulated organisation’. However, there are exceptions to the rules. For instance, only designated FDA employees, called ‘confidential filers’, are required to disclose conflicts of interest. Employees that are not required to disclose their financial conflicts of interest may hold financial interests up to US$15,000 under certain conditions (FDA, 2017*a*). We found some information relevant to statements (2) and (3) in the FDA Manuals of Policies and Procedures (MAPPs) ‘Developing and Issuing Guidance’ (FDA, 2005) and ‘Developing indication-specific Guidances’ (FDA, 2014), specifically that offices and divisions are responsible for writing the Guidances (FDA, 2014). See correspondence with the FDA in Supplement 2.

### Guideline development

EMA and FDA produce their research guidelines in a similar fashion, summarised in Table 2.

**Table 2.** Overview of commenting phases on regulatory guidelines

Guideline development steps	EMA	FDA
Announcement of concept/draft document	On the ‘Open consultations’ website (EMA, 2020*c*)	In the *Federal Register* (Federal Register, 2020) and the ‘Newly Added Guidance Documents’ website (FDA, 2020*a*)
Time period for commenting on drafts	3–6 months	2 months
Announcement of final guideline	On the ‘Open consultations’ website (EMA, 2020*c*)	In the *Federal Register* (Federal Register, 2020) and the ‘Newly Added Guidance Documents’ website (FDA, 2020*a*)
Time period for commenting on final guidelines	Always open for comments	Always open for comments
Active recruitment of stakeholders (on any step in the development)	Yes, according to guideline (but uncertain when and how)	Yes, but not necessarily
Who can comment on documents	Everybody can comment	Everybody can comment
Legislation/guidelines	Procedure for European Union guidelines and related documents within the pharmaceutical legislative framework (EMA, 2009)	Code of Federal Regulation, title 21, part 10, section 10.115, Good Guidance Practices (CFR, 2019)

Exact references and verbatim statements can be found in the full dataset (https://osf.io/3xcdu/?view_only=957edc6293894497ba7aaf5bb8fc8205).

#### EMA guidelines

The development of EMA's Clinical Efficacy and Safety Guidelines is primarily described in the guideline ‘Procedure for European Union guidelines and related documents within the pharmaceutical legislative framework’ (EMA, 2009), to which stakeholder comments have also been published (EMA, 2005). There are three stages where stakeholders may comment on the guidelines during the development: (1) first, a concept paper, which outlines the overall purpose of the guideline, is made available for commenting for 2–3 months. It is unclear if, and when, a concept paper is written with input from outside stakeholders or comments are actively solicited (EMA, 2009; section 4.4); (2) then a draft guideline is made available for comments, usually for 3–6 months. Also for this stage, it is unclear if and when comments are actively solicited (EMA, 2009; section 4.6); finally (3) the adopted guideline is published and may be commented on at any given time. EMA announces all documents for public consultation on a designated website called ‘Open consultations’ (EMA, 2020*c*).

#### FDA Guidance Documents

The Guidance development is described in the FDA Manual of Policies and Procedures ‘Developing and Issuing Guidance’ (FDA, 2005) and ‘Developing indication-specific Guidances’ (FDA, 2014), the Code of Federal Regulation, Part 10, Section 10.115 (CFR 2019), and FDA's report on Guidance Documents (FDA, 2011). There are two stages, possibly three if the draft development is included, where stakeholders may comment on Guidance Documents: (1) First, FDA develops a draft Guidance. This can be done in collaboration with individuals or organisations outside FDA, e.g. through input from workshops or Advisory Committees. Stakeholders may also submit draft proposals directly to FDA; (2) a draft Guidance Document is made publicly available for comments usually for 60 days through announcement (called a ‘notice of availability’) in the *Federal Register* (Federal Register, 2020), the official journal of the United States Government, and a designated website (FDA, 2020*a*); (3) the final Guidance Document is published and announced in the Federal Register and online and may be commented on at any given time. Every year, FDA also publishes a ‘Guidance Agenda’, a list of guidances they plan to publish or revise during the year (FDA, 2020*b*).

### Stakeholder comments

There were stakeholder comments available for six of the 13 EMA research guidelines and four of the five FDA guidelines. Seventy stakeholders submitted a total of 1014 comments, of which 947 comments could be assigned to a stakeholder. For 67 comments the stakeholder was unknown, as EMA's post-traumatic stress disorder (PTSD) guideline did not assign the individual comments to the stakeholder. There were 52 unique stakeholders of which two, International Society for CNS Clinical Trials and Methodology and Lundbeck, commented on both EMA and FDA documents. See the full dataset for details.

#### EMA guidelines

Thirty-eight stakeholders made 771 comments on six EMA guidelines. There were 25 unique stakeholders; 19 stakeholders commented on one guideline and six stakeholders commented on two or more guidelines, Lundbeck and the European Federation of Pharmaceutical Industries and Associations (five guidelines), Merck Sharp & Dome (three), and European College of Neuropsychopharmacology, International Federation of Association of Pharmaceutical Physicians, and Hoffman-La Roche (two each). We categorised 25 (66%) stakeholders as ‘industry’, nine (24%) as ‘not-industry but with industry-related conflicts’ and four (10%) as ‘independent’ (Table 2). On the comment level, we categorised 534 (76%) comments as ‘industry’, 148 (21%) as ‘not industry but with industry-related conflicts’, 22 (3%) as ‘independent’ and 67 were not categorised (Table 3).

**Table 3.** Stakeholders categorised by conflicts of interest

Regulator	Guideline	No. stakeholders	‘Industry’ (%)	‘Not-industry but with industry-related conflicts’ (%)	‘Independent’ (%)	‘Unclear’ (%)
EMA	ADHD	10	6 (60%)	4 (40%)	0	0
EMA	Schizophrenia	7	5 (71%)	2 (29%)	0	0
EMA	Alcohol dependence	6	4 (67%)	1 (17%)	1 (17%)	0
EMA	PTSD	5	4 (80%)	0	1 (20%)	0
EMA	Insomnia	5	3 (60%)	2 (40%)	0	0
EMA	Premenstrual dysphoric disorder	5	3 (60%)	0	2 (40%)	0
**EMA subtotal**		**38**	**25 (66**%**)**	**9** (**24**%**)**	**4** (**10**%**)**	**0**
FDA	ADHD	7	2 (29%)	3 (43%)	0	2 (29%)
FDA	Opioid substance disorder	3	2 (67%)	0	0	1 (33%)
FDA	Major depressive disorder	17	6 (35%)	5 (29%)	2 (12%)	4 (24%)
FDA	Alcoholism	5	3 (60%)	1 (20%)	0	1 (20%)
**FDA subtotal**		**32**	**13 (41**%**)**	**9** (**28**%**)**	**2** (**6**%**)**	**8 (25**%**)**
Total		70	38 (54%)	18 (26%)	6 (9%)	8 (11%)

#### FDA Guidance Documents

Thirty-two stakeholders made 243 comments on four FDA Guidance Documents. There were 29 unique stakeholders; 26 stakeholders commented on one document and three stakeholders, Lundbeck, Anthem Inc., and International Society for CNS Clinical Trials and Methodology, commented on two Guidance Documents each. We categorised 13 (41%) stakeholders as ‘industry’, nine (28%) as ‘not-industry but with industry-related conflicts’, two (6%) as ‘independent’ and eight (25%) as ‘unclear’ (Table 2). On the comment level, we categorised 106 comments (44%) as ‘industry’, 95 (39%) as ‘not-industry but with industry-related conflicts’, 22 (9%) as independent and 20 (8%) as ‘unclear’ (Table 4).

**Table 4.** Stakeholder comments categorised by conflicts of interest

Regulator	Guideline	No. comments	‘Industry’ (%)	‘Not-industry but with industry-related conflicts’ (%)	‘Independent’ (%)	‘Unclear’ (%)
EMA	ADHD	187	153 (82%)	34 (18%)	0	0
EMA	Schizophrenia	225	182 (80%)	43 (19%)	0	0
EMA	Alcohol dependence	99	69 (70%)	22 (22%)	8 (8%)	0
EMA	PTSD	67^a^	N/A	N/A	N/A	N/A
EMA	Insomnia	151	102 (68%)	49 (32%)	0	0
EMA	Premenstrual dysphoric disorder	42	28 (67%)	0	14 (33%)	0
**EMA subtotal**		**771 (704 assigned comments)**	**534 (76**%**)**^b^	**148** (**21**%**)**^b^	**22** (**3**%**)**^b^	**0**
FDA	ADHD	49	18 (37%)	25 (51%)	0	6 (12%)
FDA	Opioid substance disorder	21	20 (95%)	0	0	1 (5%)
FDA	Major depressive disorder	159	60 (38%)	69 (43%)	22 (14%)	8 (5%)
FDA	Alcoholism	14	8 (57%)	1 (7%)	0	5 (36%)
**FDA subtotal**		**243**	**106 (44**%**)**	**95** (**39**%**)**	**22** (**9**%**)**	**20 (8**%**)**
Total		1014 (947 assigned comments)	640 (68%)^b^	243 (26%)^b^	44 (5%)^b^	20 (2%)^b^

a The comments were not assigned to the individual stakeholders.

b Calculated as the proportion of assigned comments, not the total number of comments.

### Trial design recommendations

The EMA guidelines provided recommendations for all five trial characteristics, i.e. trial duration, psychiatric comorbidity, ‘enriched design’, efficacy outcomes, and choice of comparator in all guidelines, except ‘enriched design’ (alcohol dependence) and psychiatric comorbidity (bipolar disorder). None of the five FDA Guidance Documents contained recommendations for all five trial characteristics; for example, previous exposure to relevant medications was described in one guideline only (opioid use disorder). The trial recommendations for each guideline are summarised in Supplement 1, eTable 1–15.

#### Overlapping guidelines

EMA and FDA published overlapping guidelines on ADHD (eTable 3), depression (eTable 5), and alcohol dependence (eTable 10). The three FDA guidelines were draft versions and did not contain recommendations on ‘enriched design’ or on psychiatric comorbidity in the ADHD guideline. The overlapping guidelines concurred on trial durations for depression and alcohol dependence, e.g. EMA recommended 4–8 weeks and FDA 6–8 weeks in depression trials, and both agencies recommended a 6-month randomised withdrawal design to assess long-term outcomes. The EMA guidelines seemed more restrictive regarding psychiatric comorbidity, whereas the FDA depression and alcohol dependence guidelines emphasised the inclusion of relevant comorbidity. The guidelines agreed on the use of rating scales in ADHD and depression trials as efficacy outcomes and on the use of full abstinence or no heavy drinking for alcohol dependence.

#### Trial duration

EMA recommended short- and long-term trial durations in all 12 available guidelines. Short-term durations ranged from 2 to 14 weeks, with the exception of alcohol dependence (3–6 months) and premenstrual dysphoric syndrome (6 months). Long-term durations ranged from 3 to 15 months, of which seven guidelines recommended the randomised withdrawal design, three guidelines (ADHD, insomnia, alcohol dependence) recommended a placebo-controlled trial or the randomised withdrawal design, and two guidelines did not recommend any particular design (bipolar disorder and premenstrual dysphoric disorder).

The FDA recommendations were more varied; 6–8 weeks for depression, 24 weeks for ‘low sexual interest and/or arousal in women’, 6 months for alcohol dependence, and for ADHD they only gave a recommendation for long-term safety trials of 12 months to assess the effect on growth in children.

#### Psychiatric comorbidity

EMA listed extensive exclusion criteria related to psychiatric comorbidity in ten of the 12 available guidelines. Two guidelines (bipolar disorder and depression) did not specify exclusion criteria.

The three FDA Guidance Documents (depression, alcohol dependence, ‘low sexual interest and/or arousal in women’) that contained recommendations on psychiatric comorbidity were less restrictive than EMA's guidelines and stated that unnecessary restrictions should be avoided.

#### Enriched design

All EMA guidelines, except alcohol dependence, specified that participants could have been exposed to relevant medication prior to enrolment and there should be a ‘washout’ of such drugs.

Only one FDA Guidance Document (opioid use disorder) referred to previous exposure by stating, ‘patients should be either new entrants to treatment or stable on other treatments’.

#### Efficacy outcomes

Ten EMA guidelines recommended symptom-rating scales as efficacy measures. The alcohol dependence guideline recommended full abstinence or no heavy drinking, and the insomnia guideline recommended self-rated sleep, polysomnography, and quality of life. Several guidelines stipulated the use of ‘responder analyses’, i.e. dichotomising the participants as ‘responders’ or ‘non-responders’ based on their treatment response on symptom rating scales using arbitrary thresholds.

The FDA Guidance Documents recommended rating scales for two indications (depression and ADHD), no heavy drinking or full abstinence (alcohol dependence), urine toxicology (opioid use disorder), and number of satisfying sexual events or self-rating of sexual interests, arousal, and distress (low sexual interest and/or arousal in women).

#### Choice of comparator

Ten EMA guidelines recommended three-arm trial designs with both placebo and an active comparator. Two guidelines (alcohol dependence and premenstrual dysphoric syndrome) recommended a two-arm placebo design, but also mentioned three-arm designs as a viable option.

Four FDA Guidance Documents recommended a two-arm design with placebo (ADHD, depression, low sexual interest and/or arousal in women, and alcohol abuse), and one guideline recommended either placebo or an active comparator (opioid use disorder). The alcohol abuse guideline also mentioned the possibility of a three-arm trial design.

## Discussion

### Principal findings

To the best of our knowledge, this is the first study to systematically assess the development and content of regulatory research guidelines for psychiatric drugs. Some EMA trial design recommendations have previously been assessed, mainly focusing on the choice of comparator (Barbui and Bighelli, 2013). These regulatory guidelines are intended for industry, but the dearth of non-industry stakeholders was surprising considering the guideline process being apparently open to public comments. The FDA launched in 2010 a ‘Transparency Initiative’ (FDA, 2011) and one of the objects was to improve stakeholder involvement. FDA's stakeholder composition was indeed more varied than EMA's, although still dominated by stakeholders with conflicts of interest. EMA stated in their procedure guideline (EMA, 2009) that they would develop procedures to ensure proactive consultation of patients and patient organisations. For this cohort of guidelines, there was an absence of such stakeholders.

The guidelines recommend the design of clinical trials with limited generalisability, which may contribute to research waste (Chalmers and Glasziou, 2009). Most recommended trial durations were short and both agencies recommended the randomised withdrawal design for long-term trials. In such a trial, participants who benefit from a drug either in a placebo-controlled trial or in an open-label phase are randomised to continued active drug or placebo (FDA, 2019a; p. 18). Abrupt stopping of psychotropic drugs can lead to withdrawal symptoms, e.g. after use of antidepressants (RCP, 2019) or central stimulants, which carries an FDA black box label warning about the risk of drug dependence (FDA, 2017*b*). If these drugs are not tapered off slowly, the withdrawal symptoms may be misinterpreted as symptom deterioration and superiority of the drug over placebo. In FDA's opioid use disorder guideline it was stated, ‘Sponsors can conduct trials in patients already stable on other treatments’. This indicates that participants who are adequately treated before the trial may be randomised to placebo and thereby risk experiencing withdrawal symptoms. In EMA's schizophrenia guideline it was stated, ‘typically a few days will be appropriate’ for washing out prior antipsychotic medication during a placebo lead-in, which seems to be insufficient to avoid withdrawal symptoms.

All but two EMA guidelines allowed participants with previous exposure to the drugs to be included in the pivotal trials. Before their inclusion, such participants should ‘wash out’ the drug, i.e. stop the treatment for a certain amount of time. The sample would be ‘enriched’ because participants with a previous negative response would not be allowed to participate, either due to explicit exclusion criteria and/or because participants with a negative response would not want to participate. This trial design detail may seem trivial but it can likely lead to substantial overestimation of the benefits and underestimations of the harms.

Most EMA guidelines recommended extensive exclusion criteria regarding psychiatric comorbidity. The obsessive-compulsive disorder (OCD) guideline described that the population should be ‘pure’ OCD but the guideline also highlighted, ‘lifetime co-morbidity rates of other psychiatric diseases in patients with OCD range from 75 to 84%’. The ADHD guideline mentioned, ‘another Axis I disorder’, ‘severe comorbid symptoms such as depression and anxiety’ or ‘primary axis II disorder’ as exclusion criteria. Such exclusion criteria may reduce the trials' generalisability, e.g. most patients diagnosed with ADHD (Surman *et al*., 2010) or depression (Zimmerman *et al*., 2019) in a clinical setting are not eligible for inclusion in pivotal clinical trials. The FDA Guidance inclusion criteria were less restrictive and emphasised the inclusion of participants with comorbidities. In 2019, FDA published a draft guideline entitled ‘Enhancing the diversity of clinical trial populations’ (FDA, 2019*b*) and the Agency seems cognisant of the problem of restricted trial populations, ‘Sponsors should adopt practices for determining eligibility criteria that will allow the clinical trial population to reflect the diversity of the patients who will be using the drug if the drug is approved’ (FDA, 2019*b*).

We found that EMA and FDA mostly recommended surrogate outcomes. A recent analysis (Hey *et al*., 2020) similarly reported that 21 of 27 FDA anti-infective Guidance Documents recommended surrogate outcomes rather than patient-relevant outcomes such as mortality.

Finally, we noted that EMA mostly recommended three-arm trial designs with both placebo and active comparator. In contrast, the FDA mostly recommended two-arm placebo-controlled designs, particularly notable for ADHD and depression trials, where many approved medications are available. EMA has previously been encouraged to require comparative evidence prior to regulatory approval (Sorenson *et al*., 2011; Barbui and Bighelli, 2013; Wieseler *et al*., 2019), and such efforts seem warranted for FDA as well.

### Implication for practice and research

We identified several cases where the agencies seemed to prioritise the interests of the applicants rather than the public. For instance, FDA wrote in their opioid use disorder guideline, ‘There is great public health interest in assessing additional, clinically meaningful endpoints such as reduction in hospitalizations, emergency department visits, overdose, and death, as well as improvements in the ability to resume work, school, or other productive activity. Though understanding these outcomes would be highly valuable, the Agency recognizes that evaluating these outcomes could require larger trials than those usually conducted for marketing approval’ (FDA, 2019*c*). If the agencies wish to serve the public, the required pivotal trials should reflect the interests of the public and thus address real-world patient populations and patient-centred outcomes.

FDA informed us that their employees cannot have conflicts of interest. The FDA policy allows non-designated employees to have assets up to US$15 000 (FDA, 2017*a*). Eleven months after we submitted our request regarding access to the committee members' conflicts of interest, EMA had not processed it. It would be useful for regulatory agencies to make such information publicly available to increase transparency.

### Study limitations

The sample of guidelines was small and there was an uneven distribution of stakeholders and comments. There were challenges in counting the number of FDA comments due to the non-uniform format, and we were unable to access a few guidelines and stakeholder documents. We recognise the large variations in the stakeholder comments and that different criteria for quantifying the comments would yield different counts. However, count differences would likely be minor and unlikely to change the overall results. We were also unable to identify several of the individual commenters who submitted comments on the FDA Guidance Documents, since their affiliations were not disclosed. We also did not compare draft and final guidelines to assess if, and how, the submitted stakeholder comments might have served specific industry interests. We believed that such analyses would be too subjective.

It was a challenge to separate the stakeholders according to their conflicts of interest, especially patient groups and organisations. Some evidence points to a dose–response relationship between conflicts of interest and drug prescription patterns (Perlis and Perlis, 2016), and a recent systematic review reported that industry funding of patient organisations is common (Fabbri *et al*., 2020). We did therefore not define a lower monetary threshold, but categorised stakeholders as ‘not-industry but with industry-related conflicts’ if we identified any conflicts of interest.

## Conclusion

The EMA and FDA clinical research guidelines for psychiatric pivotal trials recommend designs that tend to have limited generalisability. Independent and non-conflicted stakeholders are underrepresented in the development phases and current guidelines emphasise trials with limited scope that may not offer much clinical value. EMA and FDA should reconsider their guideline development and find ways to promote greater involvement of the public and independent stakeholders. This may require greater advertisement of the guideline process to the public and active invitation of large numbers of scientists and organisations that are known to have no conflicts of interest to comment on the guidelines.

## Data Availability

The project protocol was made available on 27 January 2020 on MedRxiv (https://www.medrxiv.org/content/10.1101/2020.01.22.20018499v1). The full dataset is available from the Open Science Framework (https://osf.io/3xcdu/?view_only = 957edc6293894497ba7aaf5bb8fc8205). Questions regarding the dataset should be referred to the corresponding author.

## Supplementary material

For supplementary material accompanying this paper visit http://dx.doi.org/ 10.1017/S2045796021000147. click here to view supplementary material
