# Supplementary material for: EMA and FDA psychiatric drug trial guidelines: assessment of guideline development and trial design recommendations
Source: Epidemiol Psychiatr Sci. 2021 Apr 30;30:e35. doi: 10.1017/S2045796021000147 (PMC8157504; doi:10.1017/S2045796021000147)
Supplement: Supplementary file 1 [file epssup.zip › S2045796021000147sup001.docx]

**Supplement 1**

Kim Boesen, Peter C Gøtzsche, John PA Ioannidis (2021). EMA and FDA psychiatric drug trial guidelines: Assessment of guideline development and trial design recommendations

Table of Contents

eMethods 2

Inclusion of drug regulatory agency guidelines 2

Guideline development 2

Stakeholder comment documents 3

Data extraction and analysis 5

Differences between the protocol and the final version 6

eResults 7

FDA Guidance Documents 7

Guideline committee member lists 7

Guideline recommendations versus clinical context 8

eReferences 21

## eMethods

### *Inclusion of drug regulatory agency guidelines*

*EMA Clinical Efficacy and Safety Guidelines*

The EMA *Clinical Efficacy and Safety Guidelines* are available on EMA’s website. We screened all guidelines archived under the tag “Nervous system” (<https://www.ema.europa.eu/en/human-regulatory/research-development/scientific-guidelines/clinical-efficacy-safety/clinical-efficacy-safety-nervous-system>).

*FDA Guidance Documents*

FDA makes their *Guidance Documents* available on the website (<https://www.fda.gov/drugs/guidance-compliance-regulatory-information/guidances-drugs>). We screened all guidelines labelled with the tags: “drugs” (Product), “Clinical/Medical” (topic), and “Center for Drug Evaluation and Research” (FDA Organization).

*FDA File Docket Summary*

The *Docket’s Management* is available from <https://www.regulations.gov/>. To access the Guidance Documents’ Docket Folder Summaries, you click on the Docket ID number, which is reported for each Guidance Document on the FDA website. We downloaded all available documents and stakeholder comments on 5 February 2020.

### *Guideline development*

*Searching for information on EMA’s website*

On EMA’s site for ‘Clinical Efficacy and Safety Guidelines: Nervous system’ you can be directed to EMA’s main site on scientific guidelines (click on ‘related content’, not ‘topics’). On this site (<https://www.ema.europa.eu/en/human-regulatory/research-development/scientific-guidelines>) we identified two relevant documents on guideline development: “Procedure for European Union guidelines and related products within the pharmaceutical legislative framework” (<https://www.ema.europa.eu/en/documents/scientific-guideline/procedure-european-union-guidelines-related-documents-within-pharmaceutical-legislative-framework_en.pdf>) and the corresponding stakeholder comment document (<https://www.ema.europa.eu/en/documents/other/overview-comments-received-draft-guideline-procedure-eu-guidelines-related-documents-within_en.pdf>). We did not assess the underlying legislative directive, 2001/83/EC, as it seems redundant with the guideline available to sum up the guideline development.

*Searching for information on FDA’s website*

We searched for FDA Manual of Policies and Procedures (MAPPs) related to the Center for Drug Evaluation and Research (CDER) procedures on the development of Guidance Documents. There were 183 MAPPs published by to CDER (on 13 April 2020), available from: <https://www.fda.gov/about-fda/center-drug-evaluation-and-research-cder/cder-manual-policies-procedures-mapp>.

Two of these MAPPs seemed relevant: “Developing and Issuing Guidance”, March 2005 (<https://www.fda.gov/media/71702/download>), and “Developing Indication-Specific Guidances”, November 2014 (<https://www.fda.gov/media/71717/download>).

### *Stakeholder comment documents*

*FDA Guidance Documents*

We included all comments available on each Guidance Document’s Docket File Summary submitted to the draft versions, notices of availability, and final guidelines.

*Counting the number of comments in EMA and FDA stakeholder documents*

In contrast to EMA, which collects and uniformly presents the stakeholder comments in one document, FDA makes available the individual stakeholder comments in the form they were submitted to the Agency. This leads to a greater variability in the structure of the comments. To harmonise the data extraction, we applied these rules:

(1) The unit of analysis was one individual comment, which could be a section or paragraph, regarding one particular subject related to the draft or guideline.

(2) One comment could be spread over several paragraphs, provided it was related to the same issue.

(3) If the submitted comment consisted of one long paragraph with no clear separation in content, we counted this as one comment.

(4) We included initial “introductory” parts of the submitted material as a comment as well, since these “introductory” or “general overview comments” were also included from the EMA stakeholder documents. If a cover letter was accompanying a comment submitted to FDA, we tried to avoid double counting of such general comments.

(5) We did not count passages that were not related to the guideline itself, but were of more general character, such as descriptions of the submitting organisation or company in cover letters.

(6) If several comments were raised within the same box (it occurred in EMA’s stakeholder documents) and EMA gave several responses, also within the same box, we counted the number of EMA responses as the number of raised comments.

*Categorising stakeholders according to their conflicts of interest*

We categorised the stakeholders as:

(1) *Industry* (pharmaceutical companies, pharmaceutical associations, and other private companies).

(2) *Not-industry but with industry-related conflicts* (organisations, associations or individuals reporting financial conflicts of interest related to pharmaceutical companies).

(3) *Independent* (organisations, associations and individuals without conflicts of interest related to the pharmaceutical industry).

(4) *Unclear financial relationship* (there was insufficient information to identify and/or categorise the stakeholder).

If the stakeholder was an association, academic society or similar, we searched for information related to the organisation’s funding and disclosed conflicts of interest for board and/or executive members. To separate between “independent” and “not-industry but with industry-related conflicts” organisations and associations, we searched for information declared on their respective websites and in annual reports about funding from pharmaceutical companies. We also took into consideration industry sponsorships in relation to annual meetings and conferences, as substantial revenues can be gained through the hosting of such events by providing opportunities for industry to arrange “satellite educational symposia” and similar activities. We extracted all available information and made an overall assessment of the conflicts of interest. We did not define a lower monetary threshold for distinguishing between ‘not-industry but with industry related conflicts’ and ‘independent stakeholders’.

For individual stakeholders we searched on Google and PubMed to identify the commenter. If we were able to identify the stakeholder, we extracted data on declared conflicts of interest from websites, published journal articles and searched the Open Payments Data (<https://openpaymentsdata.cms.gov/>) for disclosed information about received industry payments.

We categorised drug regulatory agencies as “independent” stakeholders. This classification can be contested considering that drug regulatory agencies get substantial revenues from pharmaceutical companies for processing and approving new drug applications (EMA, 2021; FDA, 2019).

### *Data extraction and analysis*

One researcher (KB) identified the research guidelines and stakeholder comments, extracted outcome data, and looked up conflicts of interests (Feb to April 2020). Another researcher (PCG) was involved in case of doubt about the inclusion of guidelines, conflicts of interest categorisation, or stakeholder comments. We reported the total number of guidelines, year of publication, and version type; total number of guideline committee members on each guideline, and proportion of guideline committee members according to their declared conflicts of interest; total number of stakeholders, total number of stakeholder comments on each guideline, and the proportion of stakeholders and comments categorised as *industry*, *not-industry but with industry-related conflicts*, *independent*, and *unclear relationship*. We did not apply statistics on the analyses of the commenting phase and the description of the trial design recommendations.

### *Differences between the protocol and the final version*

1) We stated in our protocol (Boesen et al., 2020) that we would search for FDA stakeholder comments from the FDA Psychopharmacologic Drugs Advisory Meetings. At the protocol stage, we were unaware that FDA stakeholder comments on Guidance Documents are available from the Docket Management. In our final analysis we included the stakeholder comments available from the Docket Management.

2 In addition to searching for FDA stakeholders on Google and PubMed searches, we also searched the OpenPaymentsData registry for disclosed industry payments.

3) Following a peer reviewer suggestion, we assessed one further trial design: ‘choice of comparator’, e.g. placebo, active comparator, or both.

3) Instead of making separate searches to obtain epidemiological information on frequent comorbidities and the natural course of the symptoms as highlighted in our protocol (Boesen et al., 2020; table 1), we extracted available information reported in the guidelines. We assumed this were a less biased method.

## eResults

### *FDA Guidance Documents*

*Excluded guidelines*

We did not include these four guidelines: “antidepressant drugs – clinical evaluation” (9/1/1977), antianxiety drugs – clinical evaluation” (9/1/1977), “hypnotic drugs – clinical evaluation” (9/1/1977), and “psychoactive drugs in infants and children – clinical evaluation” (3/2/1998). These documents were not made for a particular condition.

### *Guideline committee member lists*

Summary of telephone meeting with FDA

31 March 2020 with Sudarshini Satchi, Branch Chief, Freedom of Information team.

Ms. Satchi informed us that:

1. FDA employees are not allowed to have conflicts of interests during their employment at the Agency.

2. Guidance documents are authored internally in the Agency by divisions or offices and not by individuals or sole authors. This is in contrast to the working procedure for the advisory committee guidance documents where external members are often invited to participate.

3. Since FDA employees are not allowed to have conflicts of interests and because Agency employees author the Documents without external input, the FDA is not in possession of conflict of interest disclosures related to the Guidance Documents.

We asked for written guidance on this and were referred to the CDER websites and MAPPs published on the development of Guidance Documents. See eMethods on how we identified the relevant MAPPs.

### *Guideline recommendations versus clinical context*

eTable 1. Autism Spectrum Disorder

| **Trial characteristic** | **EMA adopted guideline (EMA, 2017)** | **FDA guideline** | **Clinical context** |
| --- | --- | --- | --- |
| Trial duration | Short-term: 12 weeks or longer (section 7.3)  Long-term: Randomised withdrawal trial for long-term effects, duration not specified (section 7.4) | No guideline | Autism spectrum disorder is described as a life-long condition in the guideline (EMA 2017, executive summary) |
| Psychiatric comorbidity | Exclusion of severe comorbidity (psychosis, uncontrollable epilepsy, visual or hearing impairments) (section 5.2) |  | According to the guideline, co-morbid symptoms such as anxiety, depression, seizures, attention deficit, aggressive behaviours and sleep disorders are common (EMA 2017, section 1.2) |
| Enriched design | Yes, wash-out of prior medication (section 7.3) |  | The guideline recognises that stop of pharmacological treatment may lead to rebound and/or withdrawal symptoms (EMA 2017, section 8.2.3) |
| Efficacy outcomes | Core symptoms and functional outcomes by measurement tools or rating scales  Quality of life may be assessed (section 6) |  | We found one systematic review through COMET (**McConachie et al., 2015**). It concluded, *“Twelve tools were identified as having the strongest supporting evidence, the majority measuring autism characteristics and problem behaviour. The patchy evidence and limited scope of outcomes measured mean these tools do not constitute a ‘recommended battery’ for use. In particular, there is little evidence that the identified tools would be good at detecting change in intervention studies. The obvious gaps in available outcome measurement include well-being and participation outcomes for children, and family quality-of-life outcomes, domains particularly valued by our informants (young people with ASD and parents)”* (**McConachie et al., 2015**)*.* |
| Choice of comparator | 3-arm design with placebo and active comparator (section 7.3) |  | There are seemingly no available medications for this diagnosis.  According to the EMA guideline, “*at the time of writing there are no approved treatments for the core symptoms of ASD*” (Section 7.3) |

eTable 2. Bipolar disorder

| **Trial characteristic** | **EMA adopted guideline (EMA, 2001)*** | **FDA guideline** | **Clinical context** |
| --- | --- | --- | --- |
| Trial duration | Acute manic episodes: 3-4 weeks for short-term and 12 weeks for maintenance of effect (section 6.1)  Major depressive disorder: 6-8 weeks for short-term and 3-6 months for maintenance (section 6.3) | No guideline | According to the guideline, bipolar disorder is considered a chronic disorder (EMA 2001, introduction) |
| Psychiatric comorbidity | Exclusion criteria related to psychiatric comorbidity not specified |  | Frequent comorbidity was not specified in the guideline |
| Enriched design | Yes, wash-out of “relevant medication” (section 6) including those with “anti-manic properties” (section 6.1) |  | The guideline specified that participants need to be off medication “for a substantial period of time because of possible rebound phenomena” (EMA 2001, section 6.1) |
| Efficacy outcomes | Symptom rating scales (section 4) |  | We found one study through COMET (**Eyring et al., 2016**), which was a survey of 22 patients. The most important outcomes were severe depression, severe mania, quality of life, work/school functioning and social functioning (**Eyring et al., 2016**). |
| Choice of comparator | 3 arm design with placebo and active comparator (section 6) |  | There are available medications for this diagnosis |

**Note that we extracted the recommendations from the 2001 guideline, and not the 2016 concept paper since it did not contain specific recommendations.*

eTable 3. ADHD

| **Trial characteristic** | **EMA adopted guideline (EMA, 2010a)** | **FDA draft guideline (FDA, 2019a)** | **Clinical context** |
| --- | --- | --- | --- |
| Trial duration | Short term: 6-12 weeks  Long-term: 6 months trial, either as a placebo-controlled trial or randomised withdrawal trial (section 6.2.2) | In children, the effect on growth-change must be evaluated for 12-months. Not specified if randomised, placebo-controlled design (line 218)  Not specified for adults | According to the guideline, ADHD is considered a chronic condition (EMA 2010, section 1). |
| Psychiatric comorbidity | Exclusion of any axis I disorder, severe comorbid depressive and anxiety symptoms, axis II disorder, substance abuse (section 4.2) | Not specified | Psychiatric comorbidity is “almost inevitable” in children and “only 30 % of cases are pure ADHD”. In adults especially substance abuse, borderline and antisocial personality disorder are frequent (EMA 2010, section 1.3) |
| Enriched design | Yes, washout of relevant medication for ADHD (section 6.2.1) | Not specified | The guideline recognises that stop of pharmacological treatment may lead to rebound and/or withdrawal symptoms (EMA 2010, section 7.2.1) |
| Efficacy outcomes | Rating scales of ADHD symptoms and functional domains  (section 5.1 and 5.2) | Rating scales (line 115) and “simulated workplace environment” for trials in adults (line 123) | We did not find relevant articles in the COMET database.  The systematic review of patient preferences (**Eiring et al., 2015**) included one study with ADHD patients (**Glenngård et al., 2013**). This study surveyed 285 parents of children with ADHD, adolescents, and adults with ADHD about their treatment preferences. The surveyed adult ADHD patients preferred “function during morning and school/workday” over “function during late afternoon/evening” and “side effects” (**Glenngård et al., 2013**).  The study cited another qualitative study (**Mühlbacher et al., 2009**).^,^ which surveyed 219 ADHD patients and family members. They reported that “enabling social contacts”, “no mood swings”, and “duration of effect all day” were the most important outcomes **(Mühlbacher et al., 2009**). |
| Choice of comparator | 3-arm design with placebo and active comparator (Section 6.2.1) | 2-arm design with placebo (line 109-113) | There are available medications for this diagnosis |

eTable 4. OCD

| **Trial characteristic** | **EMA adopted guideline (EMA, 2005a)** | **FDA guideline recommendation** | **Clinical context** |
| --- | --- | --- | --- |
| Trial duration | Short term: 8 – 14 weeks (section 4.4.1)  Long-term: minimum 6-months in a randomised withdrawal study with re-randomisation of responders (section 4.4.2) | No guideline | OCD is described as a chronic condition in the EMA guideline (EMA 2005, introduction) |
| Psychiatric comorbidity | Restrictions regarding psychotic illness, bipolar disorder, borderline personality disorder, social anxiety disorder, eating disorders, substance abuse disorder and depression.  It was specified the population should be “pure” OCD (section 2.2) |  | The guideline describes that the rates of psychiatric comorbidity “range from 74 % to 85 %”, including major depressive disorder, anxiety disorders, substance abuse and eating disorders (EMA 2005, section 1.1) |
| Enriched design | Yes, “concomitant psychotropic treatment should be washed out” (section 2.2) |  | The guideline recognises that stop of pharmacological treatment may lead to rebound and/or withdrawal symptoms (EMA 2005, section 5.2.1) |
| Efficacy outcomes | Rating scales of symptoms and functional outcomes  Quality may be assessed  (section 3.1 and 3.2) |  | Through COMET, we found one ongoing study **(ICHOM)** aiming to develop a standardised set of outcomes in children and young people.  The systematic review of patient preferences did not identify studies with OCD **(Eiring et al., 2015)**. |
| Choice of comparator | 3-arm design with placebo and active comparator (Section 4.4.1) |  | There are available medications for this diagnosis |

eTable 5. Depression

| **Trial characteristic** | **EMA adopted guideline (EMA, 2013)** | **FDA draft guideline (FDA, 2018)** | **Clinical context** |
| --- | --- | --- | --- |
| Trial duration | Short term: 4-8 weeks (section 4.2.1)  Long term: 6 months as randomised withdrawal trial (section 4.2.2). Placebo-controlled trials should be limited to around 6 weeks (section 4.2.1) | Short term: 6-8 weeks (line 149)  Long term: 6 months as randomised withdrawal study (line 177) | EMA writes there is “large variance in the natural course of MDD” and that “the duration of an episode varies considerably and may be more (or less than 6 months” (EMA 2013, section 4.1.2)  Described as “cyclical” in FDA guideline (FDA 2018, line 175) |
| Psychiatric comorbidity | Exclusion criteria not specified. Stated that depression with comorbid psychiatric symptoms is not the focus of the guideline (section 2) | “Avoid unnecessary restrictions of study population” to include a broad population with e.g. comorbidity (line 229) | Anxiety disorders, bipolar disorder and other axis I disorders are mentioned as comorbid diagnoses, although the frequencies are not specified (EMA 2013, section 2 and 4.1.3) |
| Enriched design | Yes, “relevant medication has to be washed out” (section 4.2.4.2) | Not specified | The EMA guideline recognises that stop of pharmacological treatment may lead to rebound and/or withdrawal symptoms (EMA 2013, section 4.5.3.11) |
| Efficacy outcomes | Rating scales of symptoms and global assessment or social functioning (section 4.2.3) | Symptom rating scales (line 240) | In COMET we identified one ongoing study to define a core outcome set for depression in adults **(COMET, 2020a).**  The systematic review of patient preferences **(Eiring et al., 2015)** included four studies and concluded that the ability to cope with daily activities were more important than depressive symptoms. |
| Choice of comparator | 3-arm design with placebo and active comparator (section 4.1.1) | 2-arm design with placebo (section C.1) | There are available medications for this diagnosis |

eTable 6. Generalised Anxiety Disorder

| **Trial characteristic** | **EMA adopted guideline (EMA, 2005b)** | **FDA guideline recommendation** | **Clinical context** |
| --- | --- | --- | --- |
| Trial duration | Short term: minimum 8 weeks (section IV, 2.1)  Long term: 6 to 12 months as a randomised withdrawal study (section IV, 2.2) | No guideline | The EMA guidelines stated, “*The Epidemiologic Catchment Area (ECA) study found that the duration of DSM-III GAD was longer than five years in 40% of patients*” (EMA 2005, introduction) |
| Psychiatric comorbidity | Exclusion of comorbid major depression, depressive symptoms, other anxiety disorders, psychotic illness, bipolar disorder, axis II disorder, substance abuse  (section II, 2) |  | Psychiatric comorbidity is “frequently associated”, such as depression and other anxiety disorders (EMA 2005, introduction) |
| Enriched design | Yes, washout of “active agents” (section IV, 2.1) |  | The guideline recognises that stop of pharmacological treatment may lead to rebound and/or withdrawal symptoms (EMA 2005, section V.2) |
| Efficacy outcomes | Rating scales of anxiety symptoms, global assessment and/or functioning (section III, 1 and III, 2)  Quality of life may be assessed (section III, 3) |  | We did not identify relevant studies in the COMET database, nor in the systematic review of patient preferences (**Eiring et al., 2015**). |
| Choice of comparator | 3-arm design with placebo and active comparator (section IV.2.1) |  | There are available medications for this diagnosis |

eTable 7. Panic Disorder

| **Trial characteristic** | **EMA guideline recommendation (EMA, 2005c)** | **FDA guideline recommendation** | **Clinical context** |
| --- | --- | --- | --- |
| Trial duration | Link to guideline not working | No guideline | Link to guideline not working |
| Psychiatric comorbidity | Link to guideline not working |  | Link to guideline not working |
| Enriched design | Link to guideline not working |  | Link to guideline not working |
| Efficacy outcomes | Link to guideline not working |  | Link to guideline not working |
| Choice of comparator | Link to guideline not working |  | Link to guideline not working |

eTable 8. Social anxiety

| **Trial characteristic** | **EMA adopted guideline (EMA, 2006)** | **FDA guideline recommendation** | **Clinical context** |
| --- | --- | --- | --- |
| Trial duration | Short term: minimum 12 weeks (section 4.2.1)  Long term: 6-12 months as a randomised withdrawal study (section 4.2.2) | No guideline | Described as a chronic condition (EMA 2006, section 4.2.2) |
| Psychiatric comorbidity | Exclusion of major depression, depressive symptoms, anxiety disorders, schizophrenia, bipolar disorder, substance abuse, axis II disorder (section 2.2) |  | Comorbidity is frequent, “50 to 80 % of clinical patients with SAD have at least one other mental disorder”, including anxiety and depressive disorders (EMA 2006, section 1) |
| Enriched design | Yes, washout of “psychoactive compounds[s] with impact on SAD” (section 2.2 and 4.2.1) |  | The guideline recognises that stop of pharmacological treatment may lead to rebound and/or withdrawal symptoms (EMA 2006, section 5.2) |
| Efficacy outcomes | Rating scales of symptoms and global assessment (section 3.1 and 3.2)  Quality of life may be assessed (section 3.3) |  | We did not identify relevant studies in the COMET database, nor in the systematic review of patient preferences (**Eiring et al., 2015**). |
| Choice of comparator | 3-arm design with placebo and active comparator (section 4.2.1) |  | There are available medications for this diagnosis |

eTable 9. Schizophrenia

| **Trial characteristic** | **EMA adopted guideline (EMA, 2012)** | **FDA guideline recommendation** | **Clinical context** |
| --- | --- | --- | --- |
| Trial duration | Short term: 6 weeks (section 4.4.3.3)  Long term: Up to 6 months as a randomised withdrawal study or 12 months in non-inferiority active comparator trial (section 4.4.4).  Or hybrid active-comparator trial (12 months) followed by randomised withdrawal (6 months) (section 4.4.4) | No guideline | Described as a chronic condition (EMA 2012, section 4.4.4) |
| Psychiatric comorbidity | Exclusion of psychiatric comorbidity to avoid confounding, but specific diagnoses not specified (section 4.4.3.2) |  | Schizophrenia is associated with manic and depressive symptoms, anxiety, OCD, substance abuse and personality disorders (EMA 2012, section 1.1) |
| Enriched design | Yes, “prior antipsychotics should be washed out” and “typically a few days will be appropriate for run-in” [of washing out the antipsychotics during a placebo run-in]  (section 4.4.3.5) |  | The risk of withdrawal or rebound symptoms not specified in the guideline, but “withdrawal and dependence” symptoms should be assessed (EMA 2012, section 4.7.1) |
| Efficacy outcomes | Symptom rating scales (section 4.2.1) |  | Through COMET, we identified a protocol for the “PARTNERS2” project (**Keeley et al., 2015**) to develop core outcome sets for patients with bipolar and schizophrenia.  The systematic review of patient preferences (**Eiring et al., 2015**) included nine studies but reported ambiguous results regarding patient preferences, “Patients with schizophrenia tended to value disease states as higher and side effects as lower, compared to other stakeholder groups” (**Eiring et al., 2015**). |
| Choice of comparator | 3-arm design with placebo and active comparator (section 4.1.1) |  | There are available medications for this diagnosis |

eTable 10. Alcohol dependence

| **Trial characteristic** | **EMA adopted guideline (EMA, 2010b)** | **FDA draft guideline (FDA, 2015)** | **Clinical context** |
| --- | --- | --- | --- |
| Trial duration | Short term: 3-6 months  Long term: 12-15 months randomised withdrawal study of responders without treatment; or 12-15 months as an RCT (if new compound)^a^  (section 4.3.3) | Minimum 6 months as an RCT (line 153 and 185) | Described as a chronic condition (EMA 2010, executive summary) |
| Psychiatric comorbidity | In general, comorbid disorders should be excluded  Patients with “milder symptoms” may be included” (section 4.1.2.) | Comorbidity allowed, although it was not specified if it also applied to psychiatric comorbidity (line 242) | Psychiatric comorbidity is common, such as personality disorder and mood disorders (EMA 2010, introduction) |
| Enriched design | Not specified | Not specified | The risk of withdrawal or rebound symptoms were not specified in the guidelines |
| Efficacy outcomes | Full abstinence or no heavy drinking (primary outcome) (section 4.2.1)  23 potential secondary outcomes listed depending on the primary outcome (section 4.2.2) | Full abstinence or no heavy drinking (line 155) | Through COMET we identified one consensus guideline about core outcome sets (**Shorter et al., 2019**).  It reported a ranking of 90 outcomes across five different domains.  The systematic review of patient preferences (**Eiring et al., 2015**) did not identify studies with alcohol dependence. |
| Choice of comparator | 2-arm design with placebo or 3-arm design with placebo and active comparator  (section 4.3.3) | 2-arm design with placebo or 3-arm design also with active comparator | There are available medications for this diagnosis.  The EMA guideline noted, “*Currently available treatments have shown only modest and inconsistent treatment effects*” (section 4.3.3) |

a) It was not clearly described how these trials should be designed and conducted.

eTable 11. PTSD

| **Trial characteristic** | **EMA adopted guideline (EMA, 2008)** | **FDA guideline** | **Clinical context** |
| --- | --- | --- | --- |
| Trial duration | Short term: 10-12 weeks  Long-term: Randomised withdrawal trial design. Duration not specified (section 6) | No guideline | PTSD is described as a chronic condition (EMA 2008, section 6) |
| Psychiatric comorbidity | Restrictions regarding depression, other anxiety disorders, OCD, psychotic illness, bipolar disorder, axis II disorders, substance abuse (section 4) |  | Frequent comorbidity was not specified in the guideline |
| Enriched design | Yes, washout of “active agents” (section 6) |  | Rebound/withdrawal phenomena upon treatment stop are recognised in the guideline (EMA 2008, section 8) |
| Efficacy outcomes | Rating scales of symptoms and global assessment (section 5) |  | Through COMET, we identified one ongoing study (**ICHOM, 2020**) to define core outcome sets for PTSD in children and adolescents.  The systematic review on patient preferences (**Eiring et al., 2015**) did not identify studies with PTSD. |
| Choice of comparator | 3-arm design with placebo and active comparator (section 6) |  | There are available medications for this diagnosis |

eTable 12. Insomnia

| **Trial characteristic** | **EMA adopted guideline (EMA 2011a)** | **FDA guideline** | **Clinical context** |
| --- | --- | --- | --- |
| Trial duration | Short term: 2-4 weeks (section 6.2.1)  Long term: Minimum 6 months double-blind RCT or (preferably) randomised withdrawal study (section 6.2.2) | No guideline | Described as great variability across life-span (EMA 2011, introduction) |
| Psychiatric comorbidity | New drugs preferably first tested in patients with primary insomnia and not secondary insomnia (insomnia as a symptom of other disorders) (section 4.2) |  | According to the guideline comorbid psychiatric disorders are frequent (EMA 2011, section 6.3) |
| Enriched design | Yes, “wash-out” of previous treatments (section 5.2.1) |  | Rebound/withdrawal phenomena upon treatment stop are recognised in the guideline (EMA 2011, section 8.1) |
| Efficacy outcomes | Self rated sleep quality and day-time functioning  Polysomnography  Quality of life (section 5.2.2) |  | Through COMET, we found one consensus statement (**Mindell et al., 2006**) about core outcomes in children adolescents. The statement recommended polysomnography and actigraphy, and self reported sleep.  The systematic review on patient preferences (**Eiring et al., 2015**) did not identify studies with insomnia. |
| Choice of comparator | 3-arm design with placebo and active comparator (section 6.2) |  | There are available medications or this diagnosis |

eTable 13. Premenstrual dysphoric disorder

| **Trial characteristic** | **EMA adopted guideline (EMA, 2011b)** | **FDA guideline** | **Clinical context** |
| --- | --- | --- | --- |
| Trial duration | “6 cycles”, i.e. 6 months (section 4.3.3)  Long-term harms: minimum “12 cycles”, study design not specified (section 4.5.4) | No guideline | Described as a chronic condition in the guideline (EMA 2011, section 1.1 and 4.3.3) |
| Psychiatric comorbidity | Restrictions regarding axis-I or substance abuse disorders (section 4.1.2) |  | Dysthymia, major depressive disorder, and anxiety disorders are the most common axis 1 comorbidities. Rates not specified (EMA 2011, section 1.5) |
| Enriched design | Yes, “relevant medication has to be washed out” (section 4.3.2) |  | Rebound and withdrawal phenomena after stop of pharmacological treatment is recognised in the guideline (EMA 2011, section 4.5.3) |
| Efficacy outcomes | Self-rated symptoms on rating scales (section 4.1.2)  Psychological, physical and functional changes measured on rating scales (section 4.2.2) |  | Through COMET, we found one systematic review (**Budeieri et al., 1994**). It assessed available symptom rating scales and concluded that a standardised outcome set is required.  The systematic review of patient preferences (**Eiring et al., 2015**) did not include studies with premenstrual dysphoric syndrome. |
| Choice of comparator | 2-arm design with placebo or 3-arm design with placebo and active comparator  (section 4.3.2) |  | Unclear whether there are available medications for this diagnosis |

eTable 14. Opioid use disorder

| **Trial characteristic** | **EMA guideline** | **FDA final guideline (FDA, 2019b)** | **Clinical context** |
| --- | --- | --- | --- |
| Trial duration | No guideline | Not specified | Natural course not specified in the guideline |
| Psychiatric comorbidity |  | Not specified | Frequent comorbidity not specified in the guideline |
| Enriched design |  | Yes, “history of previous drug product treatment for OUD is acceptable” (Section III, B, 1) | The risk of withdrawal or rebound symptoms not specified in the guideline |
| Efficacy outcomes |  | Responder rates of opioid use measured with urine toxicology (section III, B, 2)  Applicant may propose other outcomes, such as “e.g., reduction in *craving*, improvement in sleep or mood, other patient-reported outcomes” (section 3, B, 3) | Through COMET, we identified one ongoing study (**COMET, 2020b**) to establishing core outcomes for opioid misuse intervention trials.  The systematic review of patient preferences (**Eiring et al., 2015**) did not include studies of opioid abuse. |
| Choice of comparator |  | 2-arm design with placebo or active comparator (section B, 1) | There are available medications for this diagnosis |

eTable 15. Low sexual interest, desire, and/or arousal in women

| **Trial characteristic** | **EMA guideline** | **FDA draft guideline (FDA, 2016)** | **Clinical context** |
| --- | --- | --- | --- |
| Trial duration | No guideline | 24 weeks or more (line 115) | Natural course not specified in the guideline |
| Psychiatric comorbidity |  | Exclusion criteria “should be limited” to enable a representative sample of participants (line 173) | Frequent comorbidity not specified in the guideline. It is stated that psychiatric conditions can “cause or contribute to sexual dysfunction” (line 73) |
| Enriched design |  | Not specified | The risk of withdrawal or rebound symptoms not specified in the guideline |
| Efficacy outcomes |  | Number of self-reported satisfying sexual events and self rating of sexual interests, arousal and distress (line 370)  It is stated that a patient-reported outcome (PRO) “is the most appropriate clinical outcome” (line 209) | Through COMET we found one consensus document (**Clayton et al., 2013**). It recommended self-reported scales of sexual functioning (primary) and satisfying sexual events, physiologic measures, partner assessments, and relationship satisfaction (secondary) |
| Choice of comparator |  | 2-arm with placebo (Section B) | Unclear whether there are available medications for this diagnosis.   According to the FDA Guidance Document, “*There is a medical need for development of drugs with a favorable benefit-risk profile to treat women with sexual dysfunction*.” (Line 76-77) |

## eReferences

1. **Boesen K, Gøtzsche PC, Ioannidis JPA** (2020). FDA and EMA clinical research guidelines: Assessment of trial design recommendations for pivotal psychiatric drug trials (Protocol). MedRxiv 27 January 2020. Available from: <https://www.medrxiv.org/content/10.1101/2020.01.22.20018499v1>.
2. **Budeiri DJ, Po LW, Dornan JC (1994)**. Clinical trials of treatments of premenstrual syndrome: entry criteria and scales for measuring treatment outcomes. *British Journal of Obstetrics and Gynaecology* 101:689-95.
3. **COMET (2020a)**. New methods for the development of Core Outcome Set: the example of depression. No date. Available from: <http://www.comet-initiative.org/Studies/Details/1105>.
4. **COMET (2020b)**. Establishing a core endpoints set for studies examining treatments for opioid use disorder. No date. Available from: <http://www.comet-initiative.org/Studies/Details/1128>.
5. **Clayton AH, Dennerstein L, Fisher WA, et al. (2010)**. Standards for Clinical Trials in Sexual Dysfunction in Women: Research Designs and Outcomes Assessment. *The* *Journal of Sexual Medicine* 7:541-560.
6. **Eiring Ø, Landmark BF, Aas E, Salkeld G, Nylenna M, Nytrøen K (2015)**. What matters to patients? A systematic review of preferences for medication-associated outcomes in mental disorders. *BMJ Open* 5:e007848.
7. **Eiring Ø, Nylenna M, Nytrøen K (2016)**. Patient-Important Outcomes in the Long-Term Treatment of Bipolar Disorder: A Mixed-Methods Approach Investigating Relative Preferences and a Proposed Taxonomy. *Patient* 9:91-102.
8. **EMA (2001)**. European Medicines Agency. Note for guidance on clinical investigation of medicinal products for the treatment and prevention of bipolar disorder. 26 April 2001. Available from: <https://www.ema.europa.eu/en/documents/scientific-guideline/note-guidance-clinical-investigation-medicinal-products-treatment-prevention-bipolar-disorder_en.pdf>.
9. **EMA (2005a)**. European Medicines Agency. Guideline on clinical investigations of medicinal products for the treatment of obsessive compulsive disorder. 20 Jan 2005. Available from: <https://www.ema.europa.eu/en/documents/scientific-guideline/guideline-clinical-investigation-medicinal-products-treatment-obsessive-compulsive-disorder_en.pdf>.
10. **EMA (2005b)**. European Medicines Agency. Guideline on the clinical investigation of medicinal products indicated for generalized anxiety disorder. 20 Jan 2005. Available from: <https://www.ema.europa.eu/en/documents/scientific-guideline/guideline-clinical-investigation-medicinal-products-indicated-generalised-anxiety-disorder_en.pdf>.
11. **EMA (2005c)**. European Medicines Agency. Clinical investigation of medicinal products indicated for panic disorder. Available from: <https://www.ema.europa.eu/en/clinical-investigation-medicinal-products-indicated-panic-disorder>.
12. **EMA (2006)**. European Medicines Agency. Guideline on clinical investigations of medicinal products indicated for the treatment of social anxiety disorder (SAD). 26 Jan 2006. Available from: <https://www.ema.europa.eu/en/documents/scientific-guideline/guideline-clinical-investigation-medicinal-products-indicated-treatment-social-anxiety-disorder-sad_en.pdf>.
13. **EMA (2008)**. European Medicines Agency. Guideline on the development of medicinal products for the treatment of post-traumatic stress disorder (PTSD). 24 July 2008. Available from: <https://www.ema.europa.eu/en/documents/scientific-guideline/guideline-development-medicinal-products-treatment-post-traumatic-stress-disorder-ptsd_en.pdf>.
14. **EMA (2010a)**. European Medicines Agency. Guideline on the clinical investigation of medicinal products for the treatment of attention deficit hyperactivity disorder (ADHD). 22 July 2010. Available from: <https://www.ema.europa.eu/en/documents/scientific-guideline/guideline-clinical-investigation-medicinal-products-treatment-attention-deficit-hyperactivity_en.pdf>.
15. **EMA (2010b)**. European Medicines Agency. Guideline on the development of medicinal products for the treatment of alcohol dependence. 18 Feb 2010. Available from: <https://www.ema.europa.eu/en/documents/scientific-guideline/guideline-development-medicinal-products-treatment-alcohol-dependence_en.pdf>.
16. **EMA (2011a)**. European Medicines Agency. Guideline on medicinal products for the treatment of insomnia. 17 Feb 2011. Available from: <https://www.ema.europa.eu/en/documents/scientific-guideline/guideline-medicinal-products-treatment-insomnia_en.pdf>.
17. **EMA (2011b)**. European Medicines Agency. Guideline on the treatment of premenstrual dysphoric disorder (PMDD). 21 July 2011. Available from: <https://www.ema.europa.eu/en/documents/scientific-guideline/adopted-guideline-treatment-premenstrual-dysphoric-disorder-pmdd_en.pdf>.
18. **EMA (2012)**. European Medicines Agency. Guideline on clinical investigation of medicinal products, including depot preparations in the treatment of schizophrenia. 20 Sep 2012. Available from: <https://www.ema.europa.eu/en/documents/scientific-guideline/guideline-clinical-investigation-medicinal-products-including-depot-preparations-treatment_en.pdf>.
19. **EMA (2013)**. European Medicines Agency. ) Guideline on clinical investigation of medicinal products in the treatment of depression. 30 May 2013. Available from: <https://www.ema.europa.eu/en/documents/scientific-guideline/guideline-clinical-investigation-medicinal-products-treatment-depression_en.pdf>.
20. **EMA (2017)**. European Medicines Agency. Guideline on the clinical development of medicinal products for the treatment of Autism Spectrum Disorder (ASD). 9 Nov 2017. Available from: <https://www.ema.europa.eu/en/documents/scientific-guideline/guideline-clinical-development-medicinal-products-treatment-autism-spectrum-disorder-asd_en.pdf>.
21. **EMA (2021)**. European Medicines Agency. Funding. No date. Available from: <https://www.ema.europa.eu/en/about-us/how-we-work/governance-documents/funding>.
22. **FDA (2015)**. Center for Drug Evaluation and Research. Alcoholism: Developing Drugs for Treatment Guidance for Industry. Draft guidance. Feb 2015. Available from: <https://www.fda.gov/media/91222/download>.
23. **FDA (2016)**. Center for Drug Evaluation and Research. Low Sexual Interest, Desire, and/or Arousal in Women: Developing Drugs for Treatment Guidance for Industry. Draft Guidance. Oct 2016. Available from: <https://www.fda.gov/media/100833/download>.
24. **FDA (2018)**. Center for Drug Evaluation and Research. Major Depressive Disorder: Developing Drugs for Treatment Guidance for Industry. Draft guidance. Revision 1. June 2018. Available from: <https://www.fda.gov/media/113988/download>.
25. **FDA (2019a)**. Center for Drug Evaluation and Research. Attention Deficit Hyperactivity Disorder: Developing Stimulant Drugs for Treatment Guidance for Industry. Draft guidance. May 2019. Available from: <https://www.fda.gov/media/124334/download>.
26. **FDA (2019b)**. Center for Drug Evaluation and Research. Opioid Use Disorder: Developing Depot Buprenorphine Products for Treatment Guidance for Industry. Feb 2019. Available from: <https://www.fda.gov/media/120090/download>.
27. **FDA (2020)**. US Food and Drug Administration. Fact Sheet: FDA at a Glance. 18 Nov 2020. Available from: <https://www.fda.gov/about-fda/fda-basics/fact-sheet-fda-glance>.
28. **Glenngård AH, Hjelmgren J, Thomsen PH, Tvedten T (2013)**. Patient preferences and willingness-to-pay for ADHD treatment with stimulants using discrete choice experiment (DCE) in Sweden, Denmark and Norway. *Nordic Journal of Psychiatry* 67:351-9.
29. **ICHOM (2020)**. International Consortium for Health Outcomes Measurement. Anxiety, depression, OCT and PTSD in children and young people. No date. Available from: <https://www.ichom.org/portfolio/anxiety-depression-ocd-and-ptsd-in-children-and-young-people/>.
30. **Keeley T, Khan H, Pinfold V, et al. (2015)**. Core outcome sets for use in effectiveness trials involving people with bipolar and schizophrenia in a community-based setting (PARTNERS2): study protocol for the development of two core outcome sets. *Trials* 16:47.
31. **McConachie H, Parr JR, Glod M, et al (2015)**. Systematic review of tools to measure outcomes for young children with autism spectrum disorder. *Health Technology Assessment* 19(41).
32. **Mindell JA, Emslie G, Blumer J, et al. (2006)**. Pharmacologic Management of Insomnia in Children and Adolescents: Consensus Statement. *Pediatrics* 117:e1223-32.
33. **Mühlbacher AC, Rudolph I, Lincke HJ, Nübling M (2009)**. Preferences for treatment of Attention-Deficit/Hyperactivity Disorder (ADHD): a discrete choice experiment. *BMC Health Services Research* 9:149.
34. **Shorter GW, Heather N, Bray JW, Berman AH, Giles EL (2019)**. Prioritization of Outcomes in Efficacy and Effectiveness of Alcohol Brief Intervention Trials: International Multi-Stakeholder e-Delphi Consensus Study to Inform a Core Outcome Set. *Journal of Studies on Alcohol and Drugs* 80: 299-309.
