# Supplementary material for: EMA and FDA psychiatric drug trial guidelines: assessment of guideline development and trial design recommendations
Source: Epidemiol Psychiatr Sci. 2021 Apr 30;30:e35. doi: 10.1017/S2045796021000147 (PMC8157504; doi:10.1017/S2045796021000147)
Supplement: Supplementary file 1 [file epssup.zip › S2045796021000147sup002.pdf]

Supplement 2

Regulatory correspondence

Kim Boesen, Peter C Gøtzsche, John PA Ioannidis. EMA and FDA psychiatric drug trial guidelines: Assessment of guideline development and trial design recommendations.

Content

Letter to EMA (submitted through website)	Page 3
EMA first response (email)	Page 5
Reply to EMA (email	Page 6
EMA second response (email)	Page 7
Reply to EMA	Page 9
EMA third response (email)	Page 10
Letter to FDA	Page 11
FDA confirmation of receipt (email)	Page 13
FDA first response (email)	Page 14

Authors' note

We have included all relevant emails and correspondences with the agencies. We have not included courtesy and email exchanges that were irrelevant for this research project.

Stanford, California
10 February 2020

European Medicines Agency

Freedom of Information Act request

We are currently working on a research project related to EMA and FDA regulatory research guidelines on how to design pivotal psychiatric drug trials. The protocol for our project is available here: https://www.medrxiv.org/content/10.1101/2020.01.22.20018499v1.

As part of the project, we would like to assess the complete lists of guideline committee members and their declared conflicts of interest.

We would therefore like to file a Freedom of Information Act request for access to the committee member lists and their declared conflicts of interests for the following 13 EMA Clinical Efficacy and Safety Guidelines:

EMA clinical efficacy and safety guidelines:

- Clinical development of medicinal products for the treatment of autism spectrum disorder (ASD) (https://www.ema.europa.eu/en/clinical-development-medicinal-products-treatment-autism-spectrum-disorder-asd)
- Clinical investigation of medicinal products for the treatment and prevention of bipolar disorder (https://www.ema.europa.eu/en/clinical-investigation-medicinal-products-treatment-prevention-bipolar-disorder)
- Clinical investigation of medicinal products for the treatment of attention deficit hyperactivity disorder (ADHD) (https://www.ema.europa.eu/en/clinical-investigation-medicinal-products-treatment-attention-deficit-hyperactivity-disorder-adhd)
- Clinical investigation of medicinal products for the treatment of obsessive compulsive disorder (https://www.ema.europa.eu/en/clinical-investigation-medicinal-products-treatment-obsessive-compulsive-disorder)
- Clinical investigation of medicinal products in the treatment of depression (https://www.ema.europa.eu/en/clinical-investigation-medicinal-products-treatment-depression)
- Clinical investigation of medicinal products indicated for generalised anxiety disorder (https://www.ema.europa.eu/en/clinical-investigation-medicinal-products-indicated-generalised-anxiety-disorder)
- Clinical investigation of medicinal products indicated for panic disorder (https://www.ema.europa.eu/en/clinical-investigation-medicinal-products-indicated-panic-disorder)

- Clinical investigation of medicinal products indicated for the treatment of social anxiety (https://www.ema.europa.eu/en/clinical-investigation-medicinal-products-indicated-treatment-social-anxiety)
- Clinical investigation of medicinal products, including depot preparations in the treatment of schizophrenia (https://www.ema.europa.eu/en/clinical-investigation-medicinal-products-including-depot-preparations-treatment-schizophrenia)
- Development of medicinal products for the treatment of alcohol dependence (https://www.ema.europa.eu/en/development-medicinal-products-treatment-alcohol-dependence)
- Development of medicinal products for the treatment of post-traumatic stress disorder (https://www.ema.europa.eu/en/development-medicinal-products-treatment-post-traumatic-stress-disorder)
- Medicinal products for the treatment of insomnia (https://www.ema.europa.eu/en/medicinal-products-treatment-insomnia)
- Treatment of premenstrual dysphoric disorder (https://www.ema.europa.eu/en/treatment-premenstrual-dysphoric-disorder)

In the final paper, we will report the number of committee members and the proportion of committee members with declared conflicts of interest for each research guideline. We will not report individual information, such as committee member names or names of disclosed conflicts of interest.

Please contact me if our request needs further clarification.

Sincerely,

Kim Boesen, MD
Nordic Cochrane Centre

Freedom of Information Act request: EMA's Clinical and Efficacy Guidelines - ASK-67339

Garrido-Lestache Silvia <Silvia.Garrido@ema.europa.eu> Tue, Feb 11, 2020 at 4:09 PM
To: "kb@cochrane.dk" <kb@cochrane.dk>

Dear Mr Boesen ,

Thank you for your access to documents request which has been registered under reference number ASK-67339. However, before we can start processing it, we need to clarify its scope.

Your request reads as follows:

"We would therefore like to file a Freedom of Information Act request for access to the committee member lists and their declared conflicts of interests for the following 13 EMA Clinical Efficacy and Safety Guidelines… "

-

In order to process your request, we would like you to confirm that the documents requested are the following:

* List of committee members who develop each of the guidelines listed in the scope

* The Declaration of Interest (DoI) for all the members who develop each of the guidelines listed in the scope

Kindly confirm that our understanding is correct in order to start processing the request. I look forward to hearing from you soon.

Kind regards,

Silvia Garrido-Lestache

Access to Documents

Documents Access and Publication Service

Office of the Deputy Executive Director

European Medicines Agency

Classified as internal/staff & contractors by the European Medicines Agency

This e-mail has been scanned for all known viruses by European Medicines Agency.

Freedom of Information Act request: EMA's Clinical and Efficacy Guidelines - ASK-67339

Kim Boesen <kb@cochrane.dk>
To: Garrido-Lestache Silvia <Silvia.Garrido@ema.europa.eu>

Tue, Feb 11, 2020 at 8:41 PM

Dear Silvia Garrido-Lestache,

Thank you very much for your email.

Yes, it is correctly understood that we are asking for 1) A list of committee members for each of the mentioned 13 guidelines, and 2) the declaration of interests for the committee members.

Thank you very much for your help. Please contact me if my request needs further clarification.

Sincerely,
Kim Boesen
[Quoted text hidden]
--
Kim Boesen, MD
Researcher
Nordic Cochrane Centre

[EMA Service Desk] (ASK-67339) Freedom of Information Act request: EMA's Clinical and Efficacy Guidelines

Poulimenou Despina (JIRA) <noreply@ema.europa.eu> Fri, Feb 14, 2020 at 3:27 PM
Reply-To: noreply@ema.europa.eu
To: kb@cochrane.dk

Re: EMA request reference ASK-67339

Dear Dr Boesen,

Thank you for your access to documents request.

Your request will be dealt in accordance with the principles and limits established in Regulation (EC) No 1049/2001 regarding public access to European Parliament, Council and Commission documents (the Regulation), as applicable to the European Medicines Agency (EMA) pursuant to Article 73 of Regulation (EC) No 726/2004.

Please note that EMA is currently operating within the fourth phase of its business continuity plan to ensure operational continuity during its relocation to Amsterdam. Whilst every effort is being made to process all requests as soon as possible, you should be aware that due to these exceptional circumstances from October 2019 requests cannot be processed immediately and will be dealt in a chronological order from the time they were received.

Please also note that we currently have in the system 3 requests to access documents from your firm.

As it concerns several documents, the principle set out in our policy states the Agency will apply the principle of proportionality in order to avoid the core business tasks of the Agency and its performance being jeopardised by the workload related to activities conducted by the Agency in accordance with the Regulation.

ASK-60717 will be processed first

ASK-65817 and ASK-67339 will be processed in turn one by one in this order.

Should you have any different priority order in processing them, please let us know (of note this email address is a “no-reply”) otherwise we will manage them as shown above ending with request ASK-67339.

You will be informed by an ATD coordinator when your procedure starts.

Thank you for considering the above.

Yours sincerely,
Despina Poulimenou
Documents Access and Publication Service
Stakeholders & Communication Division

European Medicines Agency
Domenico Scarlattilaan 6, 1083 HS Amsterdam, The Netherlands
Telephone: +31 (0)88 781 6000

Got a question? Ask EMA at www.ema.europa.eu/contact

This message should not be relied upon as legal advice unless it is otherwise stated. If you are not the intended recipient(s) (or authorised by an addressee who received this message), access to this e-mail, or any disclosure or copying of its contents, or any action taken (or not taken) in reliance on it is unauthorised and may be unlawful. If you have received this e-mail in error, please inform the sender immediately.

==

Original Subject: Freedom of Information Act request: EMA's Clinical and Efficacy Guidelines
Original Description: Freedom of Information Act request We are currently working on a research project related to EMA and FDA regulatory research guidelines on how to design pivotal psychiatric drug trials. The protocol for our project is available here: https://www.medrxiv.org/content/10.1101/2020.01.22.20018499v1. As part of the project, we would like to assess the complete lists of guideline committee members and their declared conflicts of interest. We would therefore like to file a Freedom of Information Act request for access to the committee member lists and their declared conflicts of interests for the following 13 EMA Clinical Efficacy and Safety Guidelines: • Clinical development of medicinal products for the treatment of autism spectrum disorder (ASD) (https://www.ema.europa.eu/en/clinical-development-medicinal-products-treatment-autism-spectrum-disorder-asd) • Clinical investigation of medicinal products for the treatment and prevention of bipolar disorder (https://www.ema.europa.eu/en/clinical-investigation-medicinal-products-treatment-prevention-bipolar-disorder) • Clinical investigation of medicinal products for the treatment of attention deficit hyperactivity disorder (ADHD) (https://www.ema.europa.eu/en/clinical-investigation-medicinal-products-treatment-attention-deficit-hyperactivity-disorder-adhd) • Clinical investigation of medicinal products for the treatment of obsessive compulsive disorder (https://www.ema.europa.eu/en/clinical-

investigation-medicinal-products-treatment-obsessive-compulsive-disorder) • Clinical investigation of medicinal products in the treatment of depression (https://www.ema.europa.eu/en/clinical-investigation-medicinal-products-treatment-depression) • Clinical investigation of medicinal products indicated for generalised anxiety disorder (https://www.ema.europa.eu/en/clinical-investigation-medicinal-products-indicated-generalised-anxiety-disorder) • Clinical investigation of medicinal products indicated for panic disorder (https://www.ema.europa.eu/en/clinical-investigation-medicinal-products-indicated-panic-disorder) • Clinical investigation of medicinal products indicated for the treatment of social anxiety (https://www.ema.europa.eu/en/clinical-investigation-medicinal-products-indicated-treatment-social-anxiety) • Clinical investigation of medicinal products, including depot preparations in the treatment of schizophrenia (https://www.ema.europa.eu/en/clinical-investigation-medicinal-products-including-depot-preparations-treatment-schizophrenia) • Development of medicinal products for the treatment of alcohol dependence (https://www.ema.europa.eu/en/development-medicinal-products-treatment-alcohol-dependence) • Development of medicinal products for the treatment of post-traumatic stress disorder (https://www.ema.europa.eu/en/development-medicinal-products-treatment-post-traumatic-stress-disorder) • Medicinal products for the treatment of insomnia (https://www.ema.europa.eu/en/medicinal-products-treatment-insomnia) • Treatment of premenstrual dysphoric disorder (https://www.ema.europa.eu/en/treatment-premenstrual-dysphoric-disorder) In the final paper, we will report the number of committee members and the proportion of committee members with declared conflicts of interest for each research guideline. We will not report individual information, such as committee member names or names of disclosed conflicts of interest. Please contact me if our request needs further clarification. Sincerely, Kim Boesen, MD Nordic Cochrane Centre

This e-mail has been scanned for all known viruses by European Medicines Agency.

29/06/2020

Subject of your enquiry:

RE: EMA request reference ASK-67570

Your question(s):

Dear Despina Poulimenou,

We submitted on 10 February 2020 a Freedom of Information Act request (ASK-67570) regarding access to the committee member lists and the corresponding declarations of interests related to 13 EMA Clinical Efficacy and Safety guidelines.

We were informed by you on 14 February that our request was placed in queue.

We would like to know the status for our request and when we can expect to receive the requested information?

Sincerely,
Kim Boesen, MD
Nordic Cochrane Centre

AskEMA - Response to ASK-71136 - RE: EMA request reference ASK-67570

1 message

AskEMA No-Reply <AskEMA.noreply@ema.europa.eu> Tue, Jun 30, 2020 at 5:11 PM
To: kb@cochrane.dk

Dear Dr Boesen,

Thank you for your message regarding the status of your request ASK-67339.

As communicated in our email of 14 February 2020, EMA is currently operating within the fourth phase of its business continuity plan to ensure operational continuity during its relocation to Amsterdam. Whilst every effort is being made to process all requests as soon as possible, from October 2019 requests cannot be processed immediately and are dealt in a chronological order from the time they were received.

This message is also publicly available on the access to documents online form page: https://www.ema.europa.eu/en/about-us/contact/send-question-european-medicines-agency.

Please rest assured that we are processing all the requests for access to documents in the queue in the order in which they were received. Because your request is still in the queue, you have not received notification that we have started processing it.

At this point in time the Agency cannot confirm any specific timelines for processing your request but when it is removed from the queue and we start dealing with it, you will receive an acknowledgement from the access to documents coordinator to whom it has been assigned.

In addition, as previously communicated, please note that we have in the system other requests from your organisation that were submitted before ASK-67339 and are currently being processed. Consequently, your request cannot be removed from the queue until the ongoing requests have been finalised.

Thank you for considering the above.

Yours sincerely,
Despina Poulimenou
Documents Access and Publication Service
Stakeholders and Communication Division

European Medicines Agency

Domenico Scarlattilaan 6, 1083 HS Amsterdam, The Netherlands
Send us a question. Go to www.ema.europa.eu/contact Telephone: +31 (0)88 781 6000

We received your question(s) on: **29/06/2020**

Subject of your enquiry: **RE: EMA request reference ASK-67570**

Your question(s):
Dear Despina Poulimenou,

We submitted on 10 February 2020 a Freedom of Information Act request (ASK-67570) regarding access to the committee member lists and the corresponding declarations of interests related to 13 EMA Clinical Efficacy and Safety guidelines.

We were informed by you on 14 February that our request was placed in queue.

We would like to know the status for our request and when we can expect to receive the requested information?

Sincerely,
Kim Boesen, MD
Nordic Cochrane Centre

This e-mail has been scanned for all known viruses by European Medicines Agency.

Nordic Cochrane Centre
Rigshospitalet, Dept. 7811
Blegdamsvej 9
2100 Copenhagen Ø, Denmark
Tel: +45 35 45 71 12
E-mail: general@cochrane.dk
www.nordic.cochrane.org

Cochrane Nordic

Trusted evidence. Informed decisions. Better health.

Copenhagen, Denmark
10 February 2020

US Food and Drug Administration

Freedom of Information Act request

We are currently working on a research project related to EMA and FDA regulatory research guidelines on how to design pivotal psychiatric drug trials. The protocol for our project is available here: https://www.medrxiv.org/content/10.1101/2020.01.22.20018499v1.

As part of the project, we would like to assess the complete lists of guideline committee members and their declared conflicts of interest.

We would therefore like to file a Freedom of Information Act request for access to the committee member lists and their declared conflicts of interests for the following five FDA Guidance Documents.

FDA Guidance Documents:

- Attention Deficit Hyperactivity Disorder: Developing Stimulant Drugs for Treatment Guidance for Industry (https://www.fda.gov/regulatory-information/search-fda-guidance-documents/attention-deficit-hyperactivity-disorder-developing-stimulant-drugs-treatment-guidance-industry)

- Opioid Use Disorder: Developing Depot Buprenorphine Products for Treatment (https://www.fda.gov/regulatory-information/search-fda-guidance-documents/opioid-use-disorder-developing-depot-buprenorphine-products-treatment)

- Major Depressive Disorder: Developing Drugs for Treatment (https://www.fda.gov/regulatory-information/search-fda-guidance-documents/major-depressive-disorder-developing-drugs-treatment)

- Low Sexual Interest, Desire, and/or Arousal in Women: Developing Drugs for Treatment Guidance for Industry (https://www.fda.gov/regulatory-information/search-fda-guidance-documents/low-sexual-interest-desire-andor-arousal-women-developing-drugs-treatment-guidance-industry)

- Alcoholism: Developing Drugs for Treatment (https://www.fda.gov/regulatory-information/search-fda-guidance-documents/alcoholism-developing-drugs-treatment)

In the final paper, we will report the number of committee members and the proportion of committee members with declared conflicts of interest for each Guidance Document. We will not report individual information, such as committee member names or names of disclosed conflicts of interest.

Please contact me if our request needs further clarification.

Sincerely,

Kim Boesen, MD
Nordic Cochrane Centre
Rigshospitalet, Dept. 7811
2100 Copenhagen Ø
Denmark
+45 35 45 71 12
kb@cochrane.dk

FDA Receipt of FOI Request

FDA_FOI@fda.gov <FDA_FOI@fda.gov> Mon, Feb 24, 2020 at 3:10 PM
To: kb@cochrane.dk

Nordic Cochrane Centre Kim Boesen

Re: Confirmation # FDA2062748
Requester Ctrl #:
In Reply refer to: 2020-1608

The Food and Drug Administration (FDA) has received your Freedom of Information Act (FOIA) request for records regarding:

committee members lists and their declared conflicts of interest for five FDA Guidance Documents

We will respond as soon as possible and may charge you a fee for processing your request. If your informational needs change, and you no longer need the requested records, please contact us to cancel your request, as charges may be incurred once processing of your request has begun. For more information on processing fees, please see http://www.fda.gov/RegulatoryInformation/FOI/FOIAFees/default.htm.

Due to an increase in the number of incoming requests, we may be unable to comply with the twenty-working-day time limit in this case, as well as the ten additional days provided by the FOIA. The actual processing time will depend on the complexity of your request and whether sensitive records, voluminous records, extensive search, and/or consultation with other HHS components or other executive branch agencies are involved. Please note that requests for medical device approval records (e.g. 510K, PMA, DEN) may take up to 18 to 24 months to process.

If you have any questions about your request, please call Rochelle A. Coleman, Information Technician at 301-796-8982 or write to us at:

Division of Freedom of Information,
U.S. Food and Drug Administration
5630 Fishers Lane, Room 1050
Rockville, MD 20857
Fax: 301-827-9267

You also have the right to seek dispute resolution services from:

FDA FOIA Public Liaison
Office of the Executive Secretariat
5630 Fishers Lane, Room 1050
Rockville, MD 20857
E-Mail: FDAFOIA@fda.hhs.gov

and/or:

Office of Government
Information Services
National Archives and Administration
8601 Adelphi Road - OGIS
College Park, MD 20740-6001
Telephone: 202-741-5770
Toll-Free: 1-877-684-6448
E-Mail: ogis@nara.gov
Fax: 202-741-5769

`Note: Do NOT reply directly to this E-mail`

RE: Your FOIA request

Satchi, Sudarshini (Darshini) <Sudarshini.Satchi@fda.hhs.gov> Mon, Mar 23, 2020 at 9:40 PM
To: "kb@cochrane.dk" <kb@cochrane.dk>

Hello,

We have received your FOIA request. Please let me know if there is a time this week that we can discuss your request. Thank you.

Darshini Satchi

Branch Chief FOI Team

Division of Information Disclosure Policy

CDER

301-796-3496

This e-mail message may contain information that is protected, privileged, or confidential, and it should not be disseminated, distributed, or copied to persons not authorized to receive such information. If you are not the intended recipient, any dissemination, distribution or copying is strictly prohibited. If you think you have received this e-mail message in error, please e-mail the sender immediately at sudarshini.satchi@fda.hhs.gov.
